# A Two-Stage Approach to Important Area Detection in Gathering Place Using a Novel Multi-Input Attention Network

**DOI:** 10.3390/s22010285

**Published:** 2021-12-31

**Authors:** Jianqiang Xu, Haoyu Zhao, Weidong Min

**Affiliations:** 1 School of Information Engineering, Nanchang University, Nanchang 330031, China; xjq@ncu.edu.cn (J.X.); zhaohaoyu@email.ncu.edu.cn (H.Z.); 2 School of Software, Nanchang University, Nanchang 330047, China; 3 Jiangxi Key Laboratory of Smart City, Nanchang 330047, China

**Keywords:** important area detection, image processing algorithm, multi-input attention network, gathering place important area detection dataset

## Abstract

An important area in a gathering place is a region attracting the constant attention of people and has evident visual features, such as a flexible stage or an open-air show. Finding such areas can help security supervisors locate the abnormal regions automatically. The existing related methods lack an efficient means to find important area candidates from a scene and have failed to judge whether or not a candidate attracts people’s attention. To realize the detection of an important area, this study proposes a two-stage method with a novel multi-input attention network (MAN). The first stage, called important area candidate generation, aims to generate candidate important areas with an image-processing algorithm (i.e., K-means++, image dilation, median filtering, and the RLSA algorithm). The candidate areas can be selected automatically for further analysis. The second stage, called important area candidate classification, aims to detect an important area from candidates with MAN. In particular, MAN is designed as a multi-input network structure, which fuses global and local image features to judge whether or not an area attracts people’s attention. To enhance the representation of candidate areas, two modules (i.e., channel attention and spatial attention modules) are proposed on the basis of the attention mechanism. These modules are mainly based on multi-layer perceptron and pooling operation to reconstruct the image feature and provide considerably efficient representation. This study also contributes to a new dataset called gathering place important area detection for testing the proposed two-stage method. Lastly, experimental results show that the proposed method has good performance and can correctly detect an important area.

## 1. Introduction

**Figure 1:**
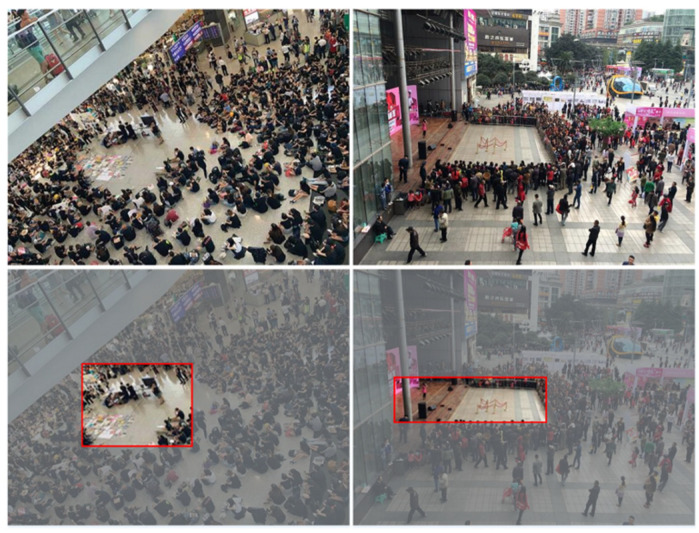
Examples of an important area in a gathering place. The first and second rows show the original images and important area with red rectangles, respectively.

An important area refers to a region that can attract people’s attention in a gathering place. People are consistently willing to considerably focus on a particular area and gather around it, such as a flexible stage, open-air dance, or some unusual event occurring in an area. Two examples of an important area where people are gathered and staring at the area are shown in Figure 1. The left side of the first row in Figure 1 shows numerous people sitting on the ground in a circle, and the right side shows a stage in the middle of the image attracting people to come and watch. The red rectangles in the second row in Figure 1 represent the important areas that this study wants to detect. The two areas attract people’s attention, and they gather around it.

Compared with the surrounding regions, an important area has evident visual features. Certain dangerous things can happen in these areas, thereby possibly affecting security. Security supervisors can locate abnormal regions by monitoring an important area. With the help of important area detection, this study can be completed automatically. However, important visual features can be easily captured by humans but not as easily for computers. Machines have difficulty understanding the semantic and context information on the importance of an area. Previous research [1,2] has proposed a vision-based model using space attention information to solve such a problem. Moreover, some studies [3] have used the graph theory and the Markov algorithm to solve the aforementioned problem. However, these methods, which are mainly based on general object detection, have failed to effectively detect important areas. The detection of an important area requires analyzing the surrounding environment and obtaining the attention information, which is difficult for traditional object detection methods.

This study proposes a two-stage method with a novel multi-input attention network (MAN) for important area detection in a gathering place. The first stage, called important area candidate generation, aims to generate candidate important areas using an image-processing algorithm. In detail, a series of image-processing algorithms is used to complete the first stage (e.g., image binarization, connected domain merging, extraction of the maximum connected domain). Candidate areas can be selected automatically for further analysis. The second stage, called important area candidate classification, aims to detect the important area from candidates with MAN. In particular, MAN is a novel classification neural network method designed as a multi-input network to give every candidate a confidence coefficient. This method can fuse an image’s global and local features to associate context information. To create the feature representation, two sub-modules (i.e., channel attention (C-A) and spatial attention (S-A) modules) are proposed based on the attention mechanism. The two sub-modules are constructed with multi-layer perceptron and pooling operation to encode the image feature. This research also contributes a new dataset, called gathering place important area detection (GPIAD), to test the efficiency and accuracy of the proposed method. This dataset contains 1200 images involving different gathering place scenes. The experimental results based on the GPIAD dataset show that the proposed approach can yield good performance.

The main contributions of this study are summarized as follows.

(1) This research proposes a novel two-stage method for important area detection with MAN. The tested results on the GPIAD dataset show the good performance of the proposed method.
(2) The first stage aims to generate candidate important areas with the combination of a series of image-processing algorithms (i.e., K-means++, image dilation, median filtering, and RLSA algorithm), which can generate high-quality results.
(3) The second stage proposes MAN, which is designed as a multi-input structure and based on an attention mechanism. The network includes the C-A and S-A modules, which are beneficial in improving the accuracy of classification.

The remainder of this paper is organized as follows. Section 2 discusses the related studies. Section 3 presents an overview of the entire method. Section 4 introduces the proposed candidate important area generation algorithm. Section 5 shows the details of the second stage and MAN. Section 6 presents the experiment result. Lastly, Section 7 provides the conclusions and future research directions.

## 2. Related Research

Important area detection is a new but not peculiar topic. Several studies have contributed to familiar task, important object detection, or salient area detection. Important object detection methods can recognize and locate important objects, mainly important people. Some researchers have also attempted to analyze the visual saliency of an object, such as the segmentation of the foreground and background. However, the importance is different from saliency. Importance is a high-level concept of social roles. Saliency is correlated but not identical to importance. People in photos may be salient but not important, important but not salient, both, or neither.

This section will introduce general object and visual saliency detections. General object detection aims to find a special object, person, animal, or building in given images or videos. Duy et al. [4] considered people who have appeared repeatedly in a certain period from large news video databases to be important. Lee et al. [5] considered the importance of objects (including area) in egocentric videos, in which important things are those with which the camera wearer has a significant interaction. General object detection methods, such as SSD [6], fast RCNN [7], and faster RCNN [8], obtained satisfactory results. With the development of deep learning [9,10,11] and detection technology, some researchers have attempted to detect important objects. For example, [12,13] studied the importance of generic object categories. Berg et al. [14] defined the importance of an object as the likelihood that it will be mentioned in a sentence written by a person describing the image. They mainly solved the problem at a category level and thought that “area” generally tends to be the most important category. Liu et al. [15] developed a convolutional neural network architecture that aggregates feature maps at different semantic levels for image representations. Zhang et al. [16] used global context information to propose a novel end-to-end trainable framework to assist the neural network in strengthening the spatial correlation between the background and foreground. Gu et al. [17] proposed explainable graph capsule networks to replace the routing part with a multi-head attention-based graph pooling approach for important object detection.

To find the important information in images, several studies [18,19,20] have investigated visual saliency. These studies have attempted to identify parts of an image that can catch an observer’s attention. Humans, special objects, and some unusual areas tend to be naturally salient contents in images. Ullah et al. [21] conducted a survey on visual saliency detection and discussed and reviewed its co-related fields, such as eye-fixation-prediction, RGBD salient-object-detection, co-saliency object detection, and video-saliency-detection models. Jiang et al. [22] studied visual saliency in group photographs and crowded scenes. They aimed to build a visual saliency model that considers the presence of faces in the image. Zhou et al. [23] proposed a quality assessment model based on visual saliency that combines chrominance and contrast perceptual factors. Li et al. [24] conducted research on the multi-scale difference of Gaussian fusion in the frequency domain and reduced the computation required in determining the proper scale of salient objects. Nasiripour et al. [25] proposed a new method to extract an object saliency map, which can integrate extracted features based on K-means singular-value decomposition. Qi et al. [26] used a graph algorithm based on the ranking method to detect and segment the most salient objects from the background, which is designed as a two-stage ranking salient object detection method. Diao et al. [27] proposed an efficient coarse object-locating method based on the saliency mechanism that can avoid an exhaustive search across the image and generate a few bounding boxes. Yu et al. [28] presented a novel computational model for object-based visual saliency, which explicitly considers connections between attention and perceptual grouping. Except for the preceding research bases, some applications based on visual saliency are also presented. Wang et al. [29] proposed a silicone mask face anti-spoofing detection method, which can compute a saliency map based on visual saliency and facial motion characteristics. He et al. [30] proposed an object recognition method based on the visual saliency mechanism for remote-sensing images, which catches the contour of objects and extracts characteristics from the background. Chao et al. [31] considered the impact of auditory information in ODVs and combined the spatial audio and visual signals to incorporate spatial–temporal visual representation in ODVs. Researchers have determined that saliency and importance have large differences [22]. At a high level, saliency concerns what draws the observer’s attention [18].

In conclusion, the general object detection methods can locate a special object in nature scenes. However, an important area is not a definite character in shape and the general object detection methods fail to find it. In other words, important object detection methods mainly detect specific people or objects that have a large difference between areas in a gathering place. Though the visual saliency detection methods can find the awareness of some objects with attention, they are mainly used to finish the segment of the foreground and background. So, the two-stage method in this paper combines the context information and the surrounding scene feature to catch the difference between an important area and other views. It is an efficient approach that considers the traditional image processing algorithm and a deep learning method.

## 3. Overview of the Two-Stage Method for Important Area Detection

To detect an important area, which is the focus of a crowd, the current research proposes a two-stage method that mainly includes two stages (i.e., important area candidate generation and important area candidate classification). The first stage can generate the candidate important areas based on a pixel-wise process. This stage mainly uses four image-processing algorithms (i.e., K-means++, image dilation, medial filtering, and RLSA algorithm). The second stage proposes the network MAN to judge which among the candidates is an important area. MAN can analyze the global and local features of an image. To significantly express the related features, the C-A and S-A modules are proposed in MAN. The two modules can focus on important area features that are useful in finding the important area. With the help of MAN, the important area can be detected from the candidate areas.

**Figure 2:**
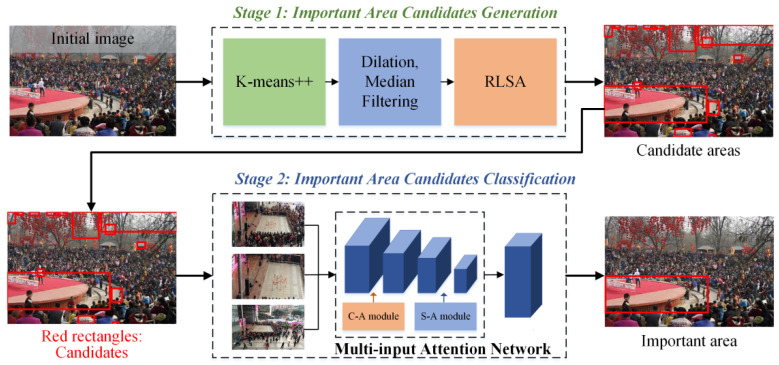
Overview of the proposed two-stage important area detection method. The method is built based on classification structure.

The entire process is presented in Figure 2. In Section 4, the candidate important areas are generated using image-processing approaches. Section 5 introduces the proposed MAN, which is used to detect an important area.

## 4. Stage One: Important Area Candidates Generation

**Figure 3:**
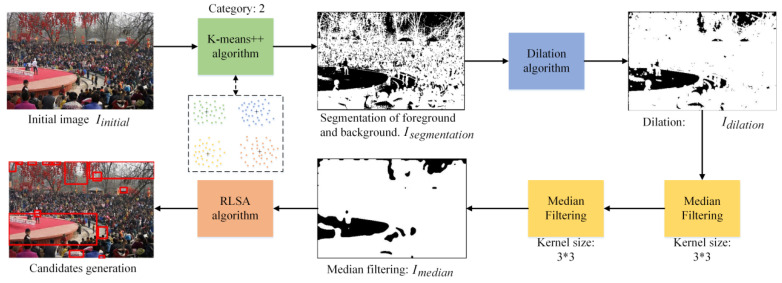
Process of the candidate important areas generation. Four traditional image-processing methods are employed, i.e., K-means++, dilation algorithm, median filtering, and the RLSA [32].

When given an image of a gathering place, this study first selects several candidates, one of which could be the important area. These candidates are processed by MAN (in Section 5) to determine which one is the important area. The entire generation process is presented in Figure 3. As shown in the original image $I_{initial}$, the important area is evidently a red stage and people surround it. $I_{initial}$ denotes the image needing to be detected.

Segmentation of the foreground and background. The K-means++ algorithm is an unsupervised clustering algorithm used for classification. To segment the foreground and background, this study presents two categories. People in a gathering place are regarded as the foreground, and the background is the candidate important areas. Thus, there are two centers of clustering $\left\{a_{1},a_{2}\right\}$ selected randomly. For each pixel data $x_{i}$, its distance to the center of clustering is calculated. Data $x_{i}$ will be divided into the class corresponding to the clustering center with the smallest distance. The new classes are represented as $\left\{C_{1},C_{2}\right\}$.

Thereafter, the two centers of clustering are recalculated using Equations (1) and (2):

(1) $$a_{1}=\frac{1}{\left|C_{1}\right|}\sum_{x\in C_{1}}x$$

(2) $$a_{2}=\frac{1}{\left|C_{2}\right|}\sum_{x\in C_{2}}x\;$$

After recalculating the two centers of clustering, the preceding step will be looped in terms of execution until the maximum iterations are reached. This study sets the maximum iterations as 10. As shown in Figure 3, the segmentation result $I_{segmentation}$ and image are dealt with through binarization. $I_{segmentation}$ represents the results of segmentation of the foreground and the background.

Binary image with dilation algorithm. The result after using the K-means++ algorithm indicates difficulty in locating the candidates. To eliminate the influence of the crowd in an image, the dilation algorithm is used for noise elimination. It can fill the hole in an object. This study attempts to locate black pixels, which represent the background. After being tested on several images, this work found that the human parts in the image will be eliminated. In the gathering place, the people are seen as the foreground and the surroundings are seen as the background. As the $I_{segmentation}$ image in Figure 3 shows, just a few outlines of people exist, and the remaining pixels mainly represent the background. The few outlines of people can further be handled by the median filtering algorithm to make the background clearer. Hence, the image with limited black pixels has superior results. If some black pixels are surrounded by white pixels, then the black pixels will be changed to white pixels. This process involves extending a boundary outward.

The result $I_{dilatioon}$ after using the dilation algorithm is shown in Figure 3. The dilation algorithm can handle the $I_{segmentation}$, then the $I_{dilatioon}$ only contains parts of the contents in $I_{segmentation}$. Most of the distractions are eliminated. Evidently, the black pixels, which represent the crowd, are eliminated. Moreover, the consecutive background parts are saved.

Median filtering algorithm. As shown in $I_{dilatioon}$ after using the dilation algorithm (see Figure 3), some small noises remain, such as impulse noise. Accordingly, the median filtering algorithm is used to remove these noises. The kernel size is chosen as 3×3. The value of the center pixel is replaced with the mid-value of the surround pixels. The mid-value of the nine pixels can be obtained via ranking. After all pixels complete this process, impulse noise can be eliminated. To ensure efficiency and a superior result, this study uses the median filtering algorithm twice. The result $I_{median}$ in Figure 3 shows that the median filtering algorithm is useful, and the majority of impulse noises are removed. The $I_{median}$ denotes the image after the processing of median filtering.

RLSA algorithm. The RLSA algorithm is used to determine the location of the candidate important area [32]. This algorithm can detect long vertical and horizontal lines, which have the same color pixels. In $I_{median}$, the candidate important area has black pixels. By using the RLSA algorithm, the neighboring black areas are linked. If two regions of black pixels are close to each other, then they are merged. This process is also in a looped execution until all black pixels are sufficiently far from others. The candidates are circled by red boxes in Figure 3. The red stage is also selected as a candidate.

**Figure 4:**
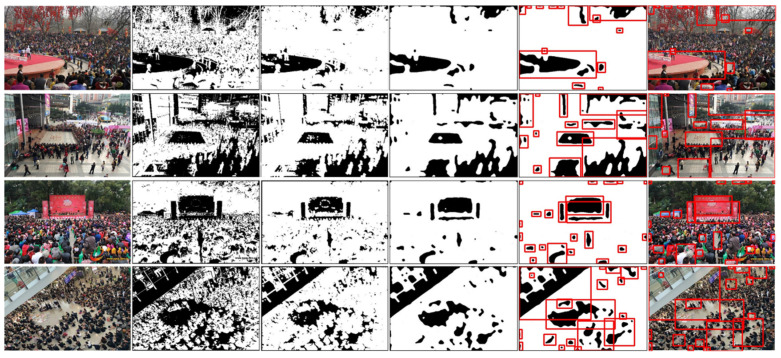
Some examples after the candidate important areas generation process.

This study also tests some other images and the results of candidate important areas, as shown in Figure 4, in which the proposed approach can select the suitable candidates. To judge which one is the true important area, this research proposes MAN, which is introduced in Section 5.

## 5. Stage Two: Important Area Candidates Classification

**Figure 5:**
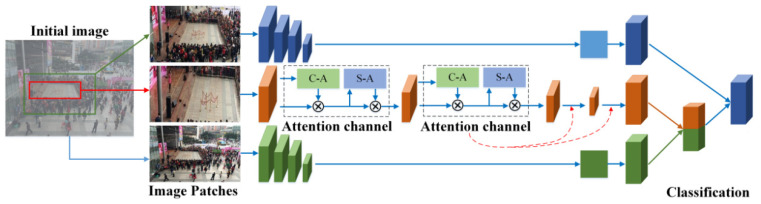
Proposed MAN.

After stage one, some candidate important areas can be determined. To judge which candidate is the important area, this stage proposes MAN based on the attention mechanism to give every candidate a confidence coefficient. MAN is designed as a multi-input structure, which is shown in Figure 5. Three types of images are sent to MAN, which represent different characteristics of an image. To improve the representation of an important area, this study utilized the attention mechanism with MAN. The C-A and S-A modules are proposed, which can focus on important features that are helpful in detecting an important area.

### 5.1. Training Images

**Figure 6:**
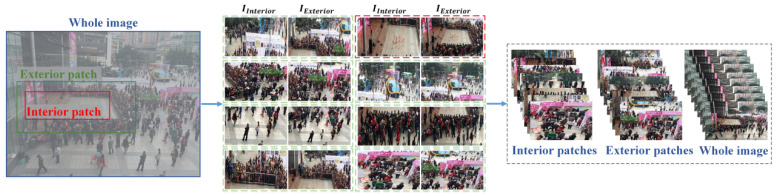
Training image patches of MAN.

To find the important area in an image, this study considers three types of images to train the model (i.e., exterior patch, interior patch, and the entire image). As shown in Figure 6, the exterior and interior patches are from the original image that can represent global and local image features. The interior patch means the area needing detection. The exterior patch means the contextual information around the area. The entire image denotes the global information of the scene.

As shown in the left image in Figure 6, the red rectangle denotes the interior patch, which mainly contains a square area. The green rectangle denotes the exterior patch, which contains the square area and also includes the people around. These people surround the important area and focus on the important area. The interior patch is obtained after the first stage of the proposed method. In the training step of CNNs, the exterior patches are obtained by hand labeling. In the test step, the exterior patches can be obtained by hand labeling or generated through the expansion of interior patch. This work tried to expand the interior patch in four edges with 10 to 100 pixels. However, the generated function is not stable due to the complex environments of crowd. This work is trying to analyze the importance of the crowd place; the extract method to generate the exterior patch automatically will be researched in the future. As shown in the middle image in Figure 6, this image has eight interior and eight exterior images. However, only one area is an important area, which is the image with the red dotted box. The other green dotted boxes show the unimportant area in the image. Hence, these areas lack crowd attention information. An observer looking at the image can easily find the area with the red dotted box and probably disregard the areas with green dotted boxes. This aspect can illustrate the significance of this study. As shown in the right image in Figure 6, the three groups of images are sent to MAN to train a model to judge the importance of the areas.

### 5.2. MAN

The proposed MAN is used to give every candidate a confidence coefficient to find the important area in a gathering place. Input images $I_{interior}$, $I_{exterior}$, and $I_{whole}$ are sent into convolutional layers. The exterior patch and the entire image are handled with a stack of convolutional layers. In stack n, the processes of $I_{exterior}$ operation can be described as Equation (3), and the processes of $I_{whole}$ operation can be described as Equation (4).

(3) $$F_{e}^{n}=ReLu(MaxPooling(Conv_{3\times 3}\left(Conv_{3\times 3}\left(I_{exterior}\right)\right)))$$

(4) $$F_{w}^{n}=ReLu(MaxPooling(Conv_{3\times 3}\left(Conv_{3\times 3}\left(I_{whole}\right)\right)))$$

In each convolutional layer, the two filters $Conv_{3\times 3}()$ of the 3×3 receptive field are used to extract the feature of an image. The max pooling operation MaxPooling() is used to reduce the size of the feature map and training parameters. After the max pooling operation, the activation function ReLu() is used to accelerate the convergence and increase the sparsity of the network. Lastly, the features $F_{e}^{n}$ and $F_{w}^{n}$ can be obtained. This study considers the six stacks of convolution layers to extract the image feature. After the final convolution layer, the features of the exterior patch $F_{e}$ and the entire image $F_{w}$ can be obtained.

Given the important function of the interior patch, which is the main character of the area, this research executes a different strategy to express its image feature. With the exception of convolutional layers, the C-A and S-A modules are utilized. When MAN is proposed, the structures of CBAM [33] have been analyzed and researched. The C-A and S-A modules are designed based on the attention mechanism. The C-A module is mainly based on channel attention and the S-A module is used to squeeze the spatial dimension of the image feature.

**Figure 7:**
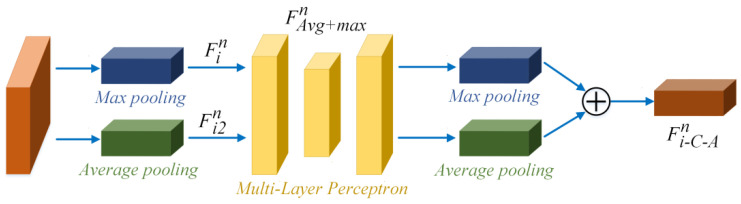
C-A module structure.

A module. The C-A module structure is shown in Figure 7. The input feature in stack n is first sent to the C-A module. The original feature is reproduced in two copies (i.e., $F_{i}^{n}$ and $F_{i2}^{n}$). The $F_{i}^{n}$ is handled with max pooling operation MaxPooling() (shows in the blue cubes in Figure 7), and $F_{i2}^{n}$ is handled with average pooling operation AvgPooling() (shows in the green cubes in Figure 7). The max pooling operation replaces the number of center pixels with the maximum value of the filter. The average pooling operation replaces the number of center pixels with an average value of the filter. After the pooling operations, the feature blocks $F_{i}^{n}$ and $F_{i2}^{n}$ can be obtained.

The features after MaxPooling() and AvgPooling() are aggregated as $F_{avg+max}^{n}$, thereby denoting average- and max-pooled features, respectively. The $F_{avg+max}^{n}$ is dealt with multi-layer perceptron (*MLP*(), shown in the yellow cubes in Figure 7), which can help to express the feature. Lastly, features after *MLP*() are merged as $F_{i-C-A}^{n}$ using element-wise summation. After the C-A module, the feature $F_{i-C-A}^{n}$ is sent to the S-A module. The preceding process can be described as Equation (5).

(5) $$F_{i-C-A}^{n}=MLP\left(MaxPooling\left(F_{i}^{n}\right)\right)+MLP\left(AvgPooling\left(F_{i2}^{n}\right)\right)$$

**Figure 8:**
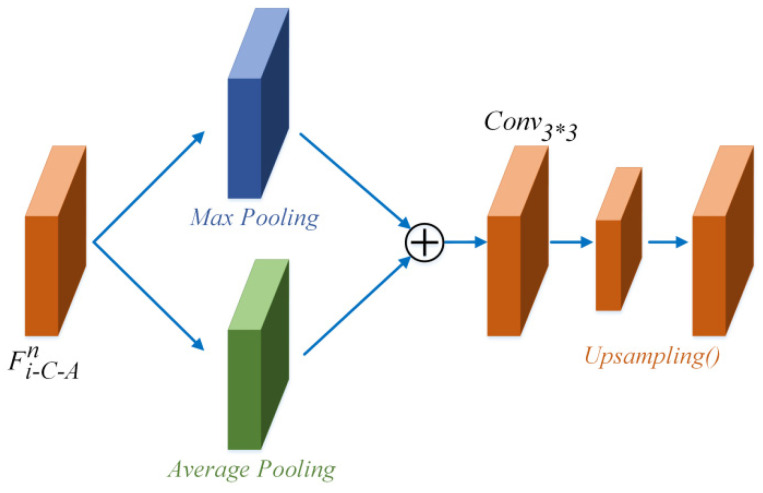
S-A module structure.

S-A module. The C-A module focuses on the available parts of the input feature, and mainly extracts the image feature channel-wise. After the C-A module, the feature $F_{i-C-A}^{n}$ is handled with the S-A module, which aims to focus on the feature spatial-wise. The S-A module structure is shown in Figure 8. The S-A module focuses on finding the region of network interest.

Feature $F_{i-C-A}^{n}$ is first handled with the max pooling operation MaxPooling() (shown in the blue cube in Figure 8) and average pooling operation AvgPooling() (shown in the green cube in Figure 8). Thereafter, the two features are concatenated and handled with a convolutional layer with a 3×3 kernel $Conv_{3\times 3}$. To maintain the size of the output, the up-sampling operation UpSampling() (shown in the orange cubes in Figure 8) is used to create the feature size after the convolutional layer. Lastly, the output $F_{i-S-A}^{n}$ can be obtained, as shown in Equation (6).

(6) $$F_{i-S-A}^{n}=UpSampling\left(Conv_{3*3}\left(MaxPooling\left(F_{i-C-A}^{n}\right)+AvgPooling\left(F_{i-C-A}^{n}\right)\right)\right)$$

The C-A and S-A modules can help MAN focus on the interior patch of the image. Such attention information is useful to recognize the important area from the candidates. The processing of C-A in Figure 5 is shown as Equation (7).

(7) $$F_{i}^{n+1}=mul\left(mul\left(F_{i}^{n},C-A\left(F_{i}^{n}\right)\right),S-A\left(mul\left(F_{i}^{n},C-A\left(F_{i}^{n}\right)\right)\right)\right)$$

The mul() operation denotes element-wise multiplication. $F_{i}^{n}$ represents the feature of the upper network and output $F_{i}^{n+1}$, which represent the important area detection results obtained via Equation (7).

After the convolutional layers, the fully connected layer is used to obtain the confidence coefficient of every input patch. The image patch with the maximum confidence coefficient is regarded as the important area.

## 6. Experiments

The proposed two-stage method for important area detection in a gathering place is implemented under the Windows 10 and Pytorch 1.2.0 experimental environment. Hardware environments are Inter Xeon E-2136 3.3 GHz and Quadro P5000. This work uses the Adam optimizer with a learning rate of 0.00001 and uses the L2 loss function to train the deep learning model. The first stage is used to select the candidate important areas, which can be processed further in the second stage with MAN. This section mainly provides the analysis of the performances of MAN in the GPIAD dataset, the visualization analysis of MAN, comparison with other SOTAs, and the related ablation studies.

### 6.1. Training Images

**Figure 9:**
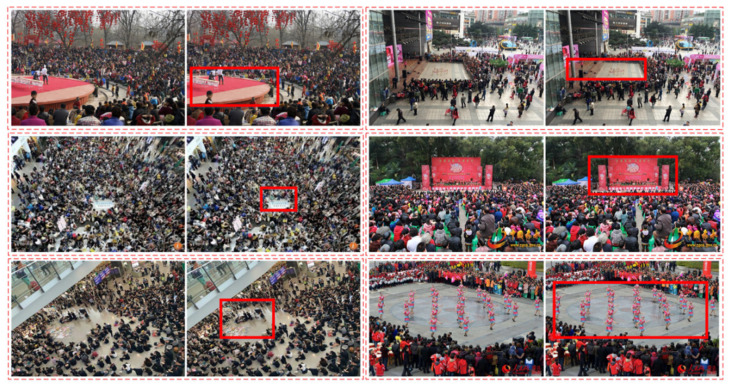
Some examples of the GPIAD dataset. In each dotted rectangle part, the left is the original image and the red full line rectangle on the right is the important area.

To prove the efficiency and accuracy of the proposed method, this study collects a new dataset called GPIAD. The self-collected dataset mainly contains 1200 gathering place scenes. Among them, the training data include 900 images, and the testing data include 300 images. Candidate areas are generated by the first stage of the proposed method. The important areas are manually annotated from these candidates. Some examples of the dataset are shown in Figure 9. In each red dotted rectangle in Figure 9, the left is the original image and the red rectangle on the right is the important area. The aim of this research is to detect the important area in images.

### 6.2. The Effect of the First Stage

In the first stage, this work proposes an important area candidate generation method with traditional image processing algorithms. This section compared different parameters with K-means++, thee dilation algorithm, and the median filtering algorithm.

**Figure 10:**
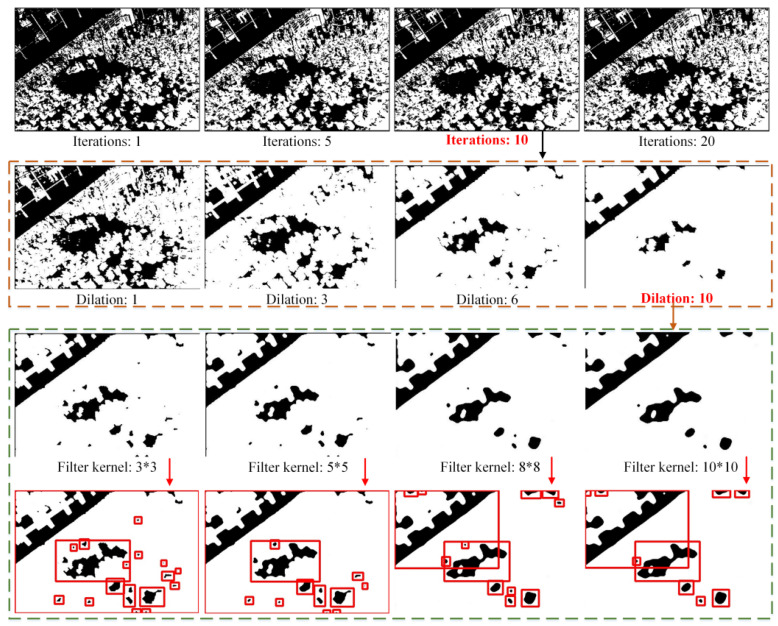
The different results with different parameters of K-means++, dilation algorithm, and the median filtering algorithm.

From Figure 10, one can observe the results of four parameters. The iteration of K-means++ chose 1, 5, 10, and 20. The dilation of the dilation algorithm chose 1, 3, 6, and 10. Lastly, the filter kernel of the median filter chose 3 × 3, 5 × 5, 8 × 8, and the 10 × 10. Different filter sizes bring different effects. This work found that when the iteration is set as 10 and the dilation is set as 10, one can obtain a good performance. Then, the media filter algorithm handles the image after these two parameters. It can be found that the 10 × 10 filter can make the edges smoother and remove some litter interferences.

### 6.3. Visualization of MAN in the GPIAD Dataset

**Figure 11:**
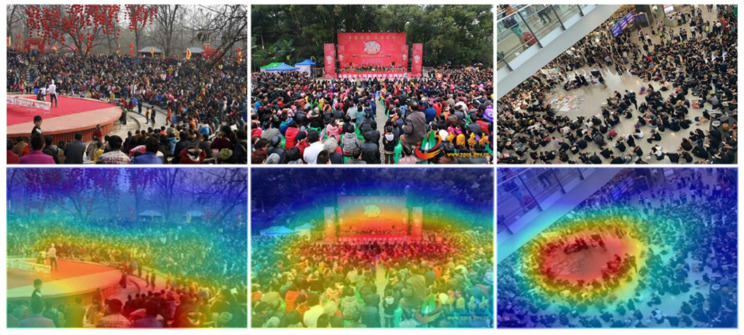
Visualization results of some images in the GPIAD dataset after MAN. The first and second lines show the nature image and results of visualization, respectively.

This research performed the visualization of the proposed MAN for important area detection. As shown in Figure 11, the first and second lines are the original images and the image with the activated part by MAN, respectively. Moreover, Figure 11 shows that MAN based on the attention mechanism can focus on the network’s attention to the important area, which is beneficial in detecting the important areas. The red regions in the second line in Figure 11 show that the important areas are activated by MAN. Compared with the blue part, the red regions are more important. The results of the visualization can prove that MAN can accurately detect the important area.

### 6.4. Performances of MAN

The MAN experiments are mainly tested in GPIAD because it contains the necessary experimental environments and data. Following the same set with [34], accuracies of Top-1 and Top-3 are calculated. An accuracy of Top-1 means the probability an important area can be detected in one candidate. An accuracy of Top-3 means the probability an important area can be detected in three candidates.

Table 1 shows that the Top-1 and Top-3 accuracies of MAN are 81.27% and 92.65%, respectively. The accuracy of Top-3 is reasonably higher than that of Top-1, which has an 11.38 difference.

This study also compares the accuracy of MAN with other classification networks, such as VGGNet [34], ResNet [35], DenseNet [36], and MobileNet [37]. These models demonstrated good performance on ImageNet, which are adapted in numerous other applications. The current research analyzes the accuracy between MAN and these networks. Table 2 shows the different models’ performances in the GPIAD dataset.

Table 2 shows that MAN obtains the best performance among these methods. MAN obtains 81.27% Top-1 accuracy and 92.64% Top-3 accuracy. VGGNet obtains 39.73% Top-1 accuracy and 58.52% Top-3 accuracy, which is lower than MAN. Top-1 accuracies of 56.12% and 65.90% are obtained by ResNet and DenseNet, respectively. The accuracy of Top-3 is clearly higher than that of Top-1. This classification framework is used to classify the different classes object, such as dog and cat, flower and tree. These objects have obvious appearance characteristics. However, when this work calls a region an ‘important area’, it is not only the area that has special appearance features, but this area also catches the most attention of crowds. So, the existing classification works fail to solve this problem well.

Note that the areas in a gathering place are enumerable and limited. MobileNet is a lightweight network structure for mobile devices. MobileNet obtains the worst results of Top-1 and Top-5, which is insufficient when used to detect important areas in a gathering place.

In addition to the classification methods, this work also compared the proposed method with several general object detection frameworks. In the training step of these methods, the initial images are used to train the model and the interior patches are set as the labels. Table 3 shows the compared mAP results in the GPIAD dataset.

From Table 3, it can be found that the detection results of general object detection methods are not satisfactory. The BBC Net obtained 28.6% mAP, M2Det obtained 32.3% mAP, YoLo v4 obtained 35.1% mAP, and this work obtained 51.4% mAP. Because the general object detection methods just detect the area through the appearance of the image, they do not consider the importance of the special area. So, the existing general object detection methods cannot be used to find the important areas directly. The proposed method employs the feature with the attention mechanism, which has advantages to finish such a task.

### 6.5. Ablation Studies

To prove the efficiency of the structure of the proposed MAN, this study conducted some ablation studies in the GPIAD dataset. Four different structures (i.e., the entire MAN, MAN without the C-A module, MAN without the S-A module, and MAN without the C-A and S-A modules) are tested, which are represented as “MAN”, “MAN-no-C-A”, “MAN-no-S-A”, and “MAN-no-CA&SA”, respectively. Table 3 shows the experimental results.

Table 4 shows that MAN obtains the best performance, which proves the efficiency of the proposed method. In addition, the performance of the “MAN-no-CA&SA” method is similar to that of VGG. The C-A and S-A modules can help the convolutional network improve the attention detection results.

## 7. Conclusions

This work proposed a two-stage method with a Multi-input Attention Network (MAN) to detect the important area in gathering place. The important area represents the most obvious region in the image that can catch most people’s attention. The candidate important areas are generated in the first stage. This work found that the background surrounding scenes can be detected by an image-processing algorithm, which are seen as the candidates in the image. The MAN is designed as a multi-input network structure based on the attention mechanism, which can fuse the global and local image features together. The C-A and S-A modules extract the feature of an important area from channel and spatial perspectives. To prove the performance of the proposed two-stage method, a new dataset, GPIAD, was collected. The experimental and ablation results show that the proposed method can obtain a good detection accuracy.

In the future work, the related method will be further researched and improved. Some new technologies, such as the Graph Convolutional Network (GCN) and the Generative Adversarial Network (GAN), are also considered to analyze this problem.

## Figures and Tables

**Table 1:** Test results with MAN in the GPIAD dataset.

Items	MAN
Top-1 Accuracy (%)	81.27%
Top-3 Accuracy (%)	92.65%

**Table 2:** Different models’ performances in the GPIAD dataset.

Methods	Top-1 Accuracy (%)	Top-3 Accuracy (%)
VGGNet [34]	39.73%	58.52%
ResNet [35]	56.12%	70.22%
DenseNet [36]	65.90%	81.15%
MobileNet [37]	38.46%	54.71%
MAN	81.27%	92.65%

**Table 3:** The comparison between the proposed method and object detection methods.

Methods	BBC Net [38]	M2Det [39]	YoLo v4 [40]	Ours
mAP	28.6%	32.3%	35.1%	51.4%

**Table 4:** Experimental results with the different structures of MAN.

Methods	Top-1 Accuracy (%)	Top-3 Accuracy (%)
MAN-no-C-A	64.25%	78.14%
MAN-no-S-A	73.46%	86.37%
MAN-no-CA&SA	39.73%	58.52%
MAN	81.27%	92.65%

## Data Availability

Not applicable.
